# Association of a Novel Protocol for Rapid Exclusion of Myocardial Infarction With Resource Use in a US Safety Net Hospital

**DOI:** 10.1001/jamanetworkopen.2020.3359

**Published:** 2020-04-22

**Authors:** Rebecca Vigen, Deborah B. Diercks, Ibrahim A. Hashim, Ambarish Pandey, Lin Zhong, Patricia Kutscher, Fernabelle Fernandez, Amy Yu, Bryan Bertulfo, Kyle Molberg, Jeffery C. Metzger, Jose Soto, Dergham Alzubaidy, Lorie Thibodeaux, Jose A. Joglar, Sandeep R. Das, James A. de Lemos

**Affiliations:** 1 Division of Cardiology, Department of Internal Medicine, University of Texas Southwestern Medical Center, Dallas; 2 Department of Emergency Medicine, University of Texas Southwestern Medical Center, Dallas; 3 Department of Pathology, University of Texas Southwestern Medical Center, Dallas; 4 Department of Population and Data Science, University of Texas Southwestern Medical Center, Dallas; 5 Rapid Response Lab, Parkland Health and Hospital System, Dallas, Texas; 6 Division of Hospitalist Medicine, Department of Internal Medicine, University of Texas Southwestern Medical Center, Dallas; 7 Quality Safety Division, Performance Improvement Department, Parkland Health and Hospital System, Dallas, Texas; 8 Center for Innovation and Value at Parkland, Parkland Health and Hospital System, Dallas, Texas

## Abstract

**Importance:**

High-sensitivity cardiac troponin T (hs-cTnT) protocols for the evaluation of chest pain in the emergency department (ED) may reduce unnecessary resource use and overcrowding.

**Objective:**

To determine whether the implementation of a novel hs-cTnT protocol, which incorporated troponin values drawn at 0, 1, and 3 hours after ED presentation and the modified HEART score (history, electrocardiogram, age, risk factors), was associated with improvements in resource use while maintaining safety.

**Design, Setting, and Participants:**

This retrospective cohort study from Parkland Health and Hospital System, a large safety net hospital in Dallas, Texas, included data on 31 543 unique ED encounters in which patients underwent electrocardiographic and troponin testing from January 1, 2017, to October 16, 2018. The hs-cTnT protocol was implemented in December 2017.

**Main Outcomes and Measures:**

Resource use outcomes included trends in ED dwell time, troponin to disposition decision time (the difference between the first troponin draw time and the time an order was placed for inpatient admission, admission to observation, or discharge), and final patient disposition. Safety outcomes included readmission for myocardial infarction and death.

**Results:**

In 31 543 encounters, mean (SD) patient age was 54 (14.4) years and 14 675 patients (48%) were female. Department dwell time decreased by a mean of −1.09 (95% CI, −2.81 to 0.64) minutes per month in the preintervention period. The decline was steeper after the intervention (−4.69 [95% CI, −9.05 to −0.33] minutes per month) (*P* for interaction = .007). The troponin to disposition time was increasing in the preintervention period by 1.72 (95% CI, 1.08 to 2.36) minutes per month; postintervention, the mean difference increased more slowly (0.37 [95% CI, −1.25 to 1.99 minutes per month; *P* value for interaction = .007]). The proportion of patients discharged from the ED increased after the intervention (48% vs 54%, *P* < .001). Thirty-day major adverse cardiac event rates were low and did not differ before and after the intervention.

**Conclusions and Relevance:**

Implementation of a novel protocol incorporating serial hs-cTnT measurements over 3 hours with the Modified HEART Score was associated with reduction in ED dwell times and attenuation of temporal increases in time from troponin measurement to disposition. This or similar protocols to rule out myocardial infarction have the potential to reduce ED overcrowding and improve health care quality while maintaining safety.

## Key Points

**Question:**

Is implementation of a novel high-sensitivity cardiac troponin T protocol in patients with chest pain associated with less resource use and within acceptable safety parameters?

**Findings:**

This cohort study of 31 543 emergency department encounters found that implementation of the protocol was associated with a shorter length of stay in the emergency department and a higher proportion of patients discharged. There was no difference in the rate of 30-day hospitalization for myocardial infarction or death.

**Meaning:**

This or similar protocols that rapidly rule out myocardial infarction have the potential to reduce emergency department overcrowding and improve health care quality.

## Teaser

This cohort study of patients at a large US safety net hospital examines the safety of and resource use associated with a new protocol for excluding myocardial infarction from diagnosis of patients with chest pains through the use of high-sensitivity troponin.

## Introduction

Emergency department (ED) overcrowding is a major public health problem in the United States and has been associated with poor outcomes, increased resource use, and restricted access to care.^1,2,3^ Chest pain is the second most common concern for patients presenting to the ED in the US, accounting for more than 7 million visits annually.^4^ In patients with suspected acute coronary syndrome (ACS), US guidelines recommend observation and serial troponin testing over 3 to 6 hours.^5^ However, the prevalence of ACS among patients with chest pain is low and decreasing over time.^6^ Prolonged observation of patients with chest pain at low risk for ACS exacerbates the problem of ED overcrowding. Streamlined protocols for the evaluation of chest pain in the ED offer the potential to improve efficiency and reduce resource use. Considering the large number of patients with chest pain evaluated annually, even small reductions in ED length of stay may have a meaningful impact on overcrowding.

A multidisciplinary team at Parkland Health and Hospital System in Dallas, Texas, and the University of Texas Southwestern Medical Center developed a novel high-sensitivity cardiac troponin T (hs-cTnT) protocol for excluding (“ruling out”) myocardial infarction (MI). This new protocol uses hs-cTnT values and changes in values at 0, 1, and 3 hours to categorize patients into 2 groups: those in whom MI can be ruled out and those with abnormal results.^7,8^ This protocol is distinct from previously studied protocols^9,10,11,12,13^ and does not leave any patients in an indeterminate zone. The protocol is integrated with the HEART score (history, electrocardiogram, age, risk factors), a validated ED risk assessment tool,^14^ to guide decisions on discharge disposition and secondary noninvasive testing. The safety and effectiveness of this novel protocol have not yet been studied beyond an initial pilot.^7^ Therefore, we performed a large retrospective study to evaluate whether the implementation of a novel hs-cTnT protocol for rapidly excluding MI was safe and was associated with changes in ED efficiency and patient disposition.

## Methods

### Study Design and Population

A retrospective cohort study design was used. This study was approved by the University of Texas at Southwestern institutional review board and the Parkland Health and Hospital System Office of Research Administration with a waiver of informed consent because the research posed minimal risk and was retrospective. This study followed the Strengthening the Reporting of Observational Studies in Epidemiology (STROBE) reporting guidelines for cohort studies.^15^ We queried the Parkland Health and Hospital system electronic medical record for all encounters from January 1, 2017, to October 16, 2018, in which patients had both ECG and troponin testing obtained within 3 hours of arrival and prior to the disposition decision, and who also did not undergo hemodialysis in the ED. At our hospital, patients with end-stage kidney disease who lack funding for dialysis present to the ED when they become symptomatic, where they undergo emergent hemodialysis based out of the ED. Patients undergoing emergent hemodialysis were excluded from this study, as their ED disposition metrics and outcomes would not be influenced by our protocol. For our resource use cohort (eFigure 1 in the Supplement), we excluded 91 encounters in which the testing was done on an outpatient basis or in day surgery, 470 encounters with missing values for time from troponin draw to disposition time, 1118 encounters in which the disposition decision time was recorded prior to a troponin draw time, and 9 redundant encounters. We excluded encounters in which disposition decision time was recorded prior to a troponin draw time, given that these were patients who would have likely been admitted irrespective of troponin testing for other reasons. Therefore, our final sample consisted of 31 543 ED encounters.

We categorized each encounter as to whether MI was ruled out as follows: in the preintervention period, we considered MI to have been ruled out if 1 or more troponin T test was obtained, and all values were less than 0.01 ng/L (to convert to micrograms per liter, multiply by 1.0), which is the lower detection limit for the fourth-generation Roche cTnT assay and approximates the 99th percentile value. In the postintervention period, we categorized hs-cTnT as per the protocol (eFigure 2 in the Supplement).^7^

### ED Efficiency and Disposition

The hs-cTnT protocol was implemented in December 2017. The protocol uses a combination of hs-cTnT values with the modified HEART score to guide clinicians regarding both disposition and secondary testing (eFigure 2 in the Supplement). We evaluated trends in ED dwell times, troponin to disposition decision time, and disposition category (discharge from ED vs admission to observation vs inpatient admission) before and after the intervention. Emergency department dwell time was abstracted from the electronic medical record, and defined as the difference between ED arrival time and ED departure time. Troponin to disposition decision time was the time difference between the time the first troponin test was drawn and the time a disposition order was placed. Disposition category included discharge from the ED, inpatient admission, or admission to observation.

### Safety Outcomes

The cohort was merged with data from the Dallas Fort Worth Hospital Council’s (DFWHC) combined data warehouse, which collects data from 97% of the North Texas health care market^16^ using name, address, medical record number, date of birth, and arrival date within a 24 hour window. The safety cohort (eFigure 1 in the Supplement) was defined by unique encounters in which the patient met criteria for exclusion of MI, as described previously. From the initial 31 543 unique ED encounters, in 14 688 MI was ruled out and the patient was discharged. Of these encounters, 13 180 were identified within the DFWHC data set. We evaluated rates of major adverse cardiac events (myocardial infarction or death) in the 30 days following the index presentation to Parkland Memorial Hospital. If the same patient had 2 encounters in a 30-day period, we only included the first encounter in the cohort. If the second encounter was an admission for MACE (major adverse cardiac event), this was considered an outcome. There were 726 recurrent encounters within 30 days of initial patient presentation for diagnoses other than MACE, and these were excluded; therefore, safety was assessed in a final cohort of 12 454 unique index encounters. Myocardial infarction was defined by a principal *International Statistical Classifiction of Diseases, Tenth Revision* (*ICD-10*) diagnosis of I21.0-3 for ST-segment elevation myocardial infarction and I21.4 for non–ST-segment elevation myocardial infarction, and revascularization was identified by *ICD-10* code 0270-0273 for percutaneous coronary intervention and code 0210-0213 for coronary artery bypass graft. Death was also collected via the DFWHC data set.

### Statistical Analysis

We evaluated temporal trends in ED dwell times, troponin to disposition decision, and final patient disposition before and after implementation of the protocol by month using an interrupted time series approach. We used linear regression models to assess changes in the continuous outcomes (ED dwell time and troponin to disposition time) and multinomial logistic regression models to assess changes in categorical outcomes (disposition status). In each linear model or multinomial logistic regression model, we included the interaction term of month and the binary variable of before vs after intervention. This was to compare the change of each study outcome per month before and after intervention. We performed these analyses in the entire sample of encounters, as well as in several predefined subsets of encounters: (1) encounters with a chief concern of chest pain listed in the electronic medical record, (2) encounters that met the definition for exclusion of MI, and (3) encounters with chest pain that met the definition for exclusion of MI. We performed both unadjusted and multivariable adjusted analyses in which we accounted for age, sex, race, ethnicity, and payment type (or *financial class*). Financial class was extracted from the electronic medical record and included Dallas County tax support, commercial insurance, Medicaid, Medicare, and self-pay (uninsured). In all these models, we added a random-effect term at the patient level to control for intrapatient effects. We also used a linear regression model to assess for the change in the intercept at the end of the preintervention period and at the beginning of the postintervention period for the continuous outcomes. These analyses were performed using R version 3.3.0 (R Project for Statistical Computing). Statistical significance was set at 2-sided *P* < .05.

## Results

The final cohort included 31 543 unique ED encounters with a mean (SD) patient age of 54 (14.4), and 14 675 (48%) were female. The population was racially and ethnically diverse with a large proportion relying on uncompensated care (Table 1). Median monthly crude ED dwell times and troponin to disposition times (eFigure 3 in the Supplement) demonstrate month-to-month variation throughout the study period.

**Table 1.** Demographic and Resource Utilization Outcomes of the Cohort

Variable	No. (%)		
	Total Cohort (n = 31 543)	Preintervention (n = 16 991)	Postintervention (n = 14 552)
Age, mean (SD), y	54 (14.4)	53.8 (14.2)	54.2 (14.6)
Sex			
Female	14 675 (47.6)	8013 (48.1)	6662 (47.0)
Male	16 180 (52.4)	8661 (51.9)	7519 (53.0)
Race			
White	17 013 (55.5)	9267 (56.0)	7746 (55.0)
Black	12 623 (41.2)	6724 (40.6)	5899 (41.9)
Other	994 (3.2)	565 (3.4)	429 (3.0)
Ethnicity			
Hispanic	11 889 (38.7)	6490 (39.1)	5399 (38.2)
Non-Hispanic	18 835 (61.3)	10 099 (60.9)	8736 (61.8)
Financial class			
Uncompensated	9235 (29.9)	5146 (30.9)	4089 (28.8)
Commercial	2089 (6.8)	1043 (6.3)	1046 (7.4)
Medicaid	5876 (19.0)	3259 (19.5)	2617 (18.5)
Medicare	6082 (19.7)	3295 (19.8)	2787 (19.7)
Self-pay	7573 (24.5)	3931 (23.6)	3642 (25.7)
Exclusion class			
Abnormal	4950 (15.7)	2563 (15.1)	2387 (16.4)
Incomplete	982 (3.1)	0	982 (6.7)
Rule out	25 611 (81.2)	14 428 (84.9)	11 183 (76.8)
Disposition			
ED discharge	16 018 (50.8)	8182 (48.2)	7836 (53.8)
Inpatient admission	8898 (28.2)	4938 (29.1)	3960 (27.2)
Observation admission	6627 (21.0)	3871 (22.8)	2756 (18.9)
ED dwell time, median (IQR), min	388 (286-568)	385 (280-581)	391 (292-556)
Troponin to disposition decision time, median (IQR), min	176 (113-253)	169 (107-257)	185 (122-269)

Abbreviations: ED, emergency department; IQR, interquartile range.

**Figure 1.**
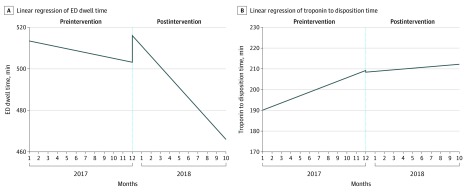
Linear Regression of Resource Use Outcomes Before and After Intervention The x-axis charts the sequence of months included in the dataset, between January 2017 and October 2018; bracketed spans mark periods before and after the intervention respectively.

When accounting for rates of change in outcomes, which isolates the association between the intervention and the outcomes, ED dwell time decreased by a mean of 1.09 (95% CI, −2.81 to 0.64) minutes per month in the preintervention period in an unadjusted model. Postintervention, dwell times decreased by 4.69 (95% CI, −9.05 to −0.33) minutes per month (*P* for interaction = .007). In the multivariable model, the mean difference in minutes per month in ED dwell time was −0.71 (95% CI, −2.49 to 1.07) before the intervention and −10.28 (−16.31 to −4.25) after the intervention (*P* for interaction <.001) (Table 2). The *P* values for interaction indicate that the changes in dwell times for both models were associated with the intervention. Similar trends were noted in the subcohorts. In the linear regression model, there was a statistically significant change in slope before vs after the intervention (*P* = .002) and no significant incremental change (*P* = .06) when comparing the intercepts at the end of the preintervention period and beginning of the postintervention period (Figure 1A).

**Table 2.** Trends in ED Dwell Time and Troponin to Disposition Decision Time

Population	Unadjusted						Adjusted^a^					
	Difference in time in ED, mean (95% CI), min/mo		*P* value^b^	Difference in time from troponin to disposition, mean (95% CI), min/mo		*P* value^b^	Difference in time in ED, mean (95% CI), min/mo		*P* value^b^	Difference in time from troponin to disposition, mean (95% CI), min/mo		*P* value^b^
	Preintervention	Postintervention		Preintervention	Postintervention		Preintervention	Postintervention		Preintervention	Postintervention	
Total cohort (n = 31 543)	−1.09 (−2.81 to 0.64)	−4.69 (−9.05 to −0.33)	.007	1.72 (1.08 to 2.36)	0.37 (−1.25 to 1.99)	.007	−0.71 (−2.49 to 1.07)	−10.28 (−16.31 to −4.25)	<.001	1.78 (1.16 to 2.4)	−0.39 (−2.5 to 1.72)	.004
Chest pain subcohort (n = 12 845)	−1.34 (−3.68 to 1)	−4.76 (−10.68 to 1.16)	.06	1.94 (1.01 to 2.87)	−0.82 (−3.17 to 1.54)	<.001	−1.2 (−3.6 to 1.2)	−9.5 (−17.68 to −1.31)	.005	2.03 (1.09 to 2.97)	−1.48 (−4.68 to 1.72)	.002
Subcohort with MI excluded (n = 25 611)	−0.75 (−2.55 to 1.05)	−3.22 (−7.86 to 1.42)	.09	1.97 (1.26 to 2.68)	−0.22 (−2.04 to 1.6)	<.001	−0.31 (−2.16 to 1.53)	−7.61 (−14.1 to −1.13)	.002	2.06 (1.38 to 2.73)	−1.15 (−3.54 to 1.24)	<.001
Chest pain and MI excluded subcohort (n = 10 917)	−0.79 (−3.13 to 1.55)	−3.06 (−9.06 to 2.95)	.23	2.14 (1.14 to 3.14)	−1.47 (−4.04 to 1.1)	<.001	−0.6 (−2.97 to 1.77)	−6.69 (−15.02 to 1.64)	.045	2.17 (1.16 to 3.18)	−2.16 (−5.71 to 1.39)	.001

^a^ Adjusted for age, race, ethnicity, and financial class.

^b^ *P* for interaction: month × intervention.

The troponin to disposition time was increasing in the preintervention period by 1.72 (95% CI, 1.08 to 2.36) minutes per month in an unadjusted model. Postintervention, the mean difference increased more slowly (0.37 minutes per month, 95% CI, −1.25 to 1.99). In the adjusted model, the mean difference in the troponin to disposition time was increasing by 1.78 (95% CI, 1.16 to 2.40) minutes per month in the preintervention period. Postintervention, the average monthly difference was decreasing by 0.39 (95% CI, −2.50 to 1.72) minutes per month. The *P* value for the interaction between month and intervention was significant for both adjusted and unadjusted models, suggesting that the differences in the period before vs after the intervention were associated with the intervention. Similar trends were seen in the subcohorts (Table 3). In the linear regression model, there was a statistically significant change in slope before vs after intervention (*P* = .007) and no significant incremental change (*P* = .07) when comparing the intercepts at the end of the preintervention period and beginning of the postintervention period (Figure 1B).

**Figure 2.**
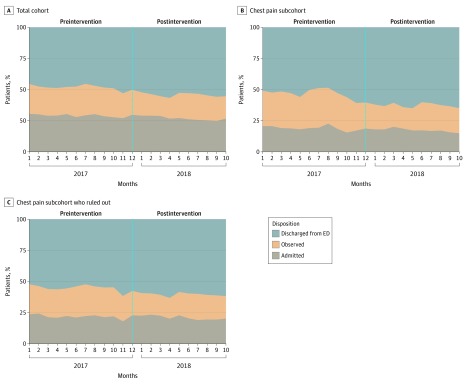
Proportion of Encounters Resulting in ED Discharge vs Observation and Admission The x-axis charts the sequence of months included in the dataset, between January 2017 and October 2018; bracketed spans mark periods before and after the intervention respectively. ED indicates emergency department.

In the 11 months before implementation of the new protocol, 48% of encounters (8181 patients) resulted in discharge from the ED with 29% (4938 patients) admitted to the hospital and 23% (3871 patients) admitted to observation. In the 10 months following the intervention, 54% (7836 patients) were discharged from the ED with lower rates of admission to the hospital or observation (*P* < .001). Monthly disposition rates for the total cohort, the subcohort with chest pain, and the subcohort with chest pain and MI excluded are shown in Figure 2. The multinomial logistic regression analyses confirm the trend analyses that are represented in the figures with overall declines in admissions throughout the study period (Table 2).

**Figure 3.**
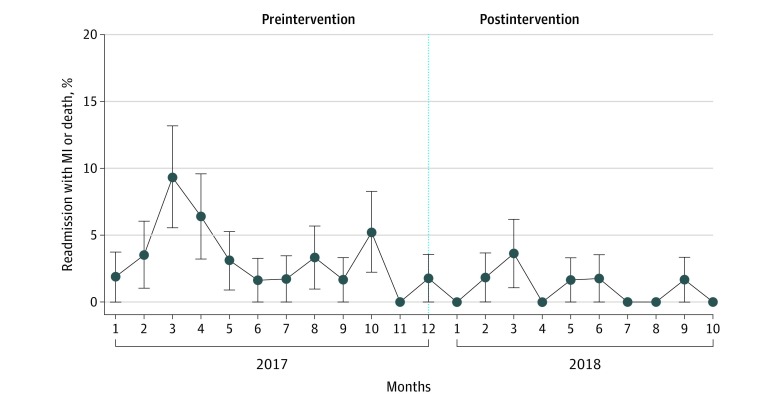
Absolute 30-Day Rates of Major Adverse Cardiac Events The x-axis charts the sequence of months included in the dataset, between January 2017 and October 2018; bracketed spans mark periods before and after the intervention respectively. Error bars represent SEs. MI indicates myocardial infarction.

Of the 12 454 unique Parkland Health and Hospital System encounters captured in the DFWHC database where MI was ruled out and patients were discharged, there were 22 deaths, 9 readmissions for MI, 5 revascularizations with percutaneous coronary intervention, and 2 revascularization with coronary artery bypass grafts in the subsequent 30 days. The absolute incidence before and after intervention were very low, less than 0.01% (Figure 3).

## Discussion

This large retrospective cohort study from one of the first US medical centers to implement the hs-cTnT assay found that implementation of a novel hs-cTnT algorithm for rapid rule out of MI was associated with favorable trends in patient disposition and ED efficiency and was not associated with increased mortality. These findings, from a large, urban safety net hospital, suggest that careful implementation of novel MI exclusion strategies with high-sensitivity troponin assays may be an effective strategy to reduce ED overcrowding.

A discrete improvement immediately upon protocol implementation was not observed for either ED dwell times or troponin-to-disposition times, as reflected by the absence of a significant fall in the intercept value. Rather, the improvements reflected modification of the slopes of temporal trends. This is likely explained by the complexity of the protocol intervention that requires extensive (and ongoing) education and continuous monitoring for quality assurance. Moreover, the protocol was implemented during December, one of the busiest and most crowded times in the ED.

Previous studies have validated different chest pain rule-out strategies, including 0-hour rule out^10,17^ and 0/1-hour rule out strategies.^11,18^ These studies have shown high negative predictive values of these strategies for rule out. However, most were carried out in non-US populations, and in these studies patient disposition was not specified by the protocol studied. Given the lower prevalence of MI in the US among populations in whom troponin testing is obtained,^7^ it is possible that use of the hs-cTnT may be associated with more false-positive MI diagnoses and subsequent hospital admissions. Therefore, careful study of hs-cTnT protocols before and after implementation is important to ensure that transition to hs-cTnT does not lead to significant increases in resource use after implementation.

Several studies have evaluated the effects of hs-cTnT algorithms on resource use.^19,20,21^ In a prospective study that evaluated the implementation of an hs-cTnT strategy combined with the HEART score, investigators found decreases in admission rates, median days in hospital, and costs.^20^ However, this study used the 99th percentile URL for the hs-cTnT and the hs-cTnI in combination with the HEART score; was conducted in Europe, where the pretest probability of MI is higher; and enrolled patients per their research protocol, which may have increased provider adherence to their algorithm. In a stepped-wedge, cluster-randomized clinical trial that allocated hospitals to implementation of an hs-cTnI (troponin I, cardiac form) assay using sex-specific 99th-percentile thresholds, rates of coronary angiography were higher in the implementation phase, while rates of percutaneous coronary intervention did not differ between baseline and implementation phase among patients reclassified by the hs-cTnI assay.^21^ The duration of hospital stay was also shorter in the implementation phase.^21^ While these studies demonstrated improvements in hospital stays using high-sensitivity troponin algorithms, they both used 99th URL cutoffs and were conducted outside of the US.

Our study is, to our knowledge, unique from prior studies in that it evaluated a novel hs-cTnT protocol that does not rely on the 99th percentile upper limit of normal of the hs-cTnT and is combined with the Modified HEART score. Additionally, it was conducted in a large cohort in a diverse, urban population in the United States. In addition, we were able to capture follow-up in most patients through matching with the Dallas Forth Worth Hospital Council Database. To our knowledge, this is the first study to demonstrate the safety of this novel hs-cTnT algorithm including the Modified HEART score.

### Limitations

There are several limitations of our study that merit acknowledgment. First, given that this was a retrospective study, we cannot completely account for secular trends in the outcomes that we observed. However, we used an interaction term to control for the intervention as well as time which suggests that the results are modified by the intervention. Second, we evaluated changes in ER dwell times, troponin to disposition decision time and final disposition, but did not evaluate costs. Third, the magnitude of change in dwell times and disposition following implementation of the protocol is of uncertain clinical and operational significance. Fourth, we assessed safety using *ICD-10* codes for myocardial infarction instead of adjudicating all cases; however, we would not expect any change in the rate of misclassification either prior or post implementation such that it is unlikely that this would affect the results. Fifth, we are unable to exclude outcome events in the unmatched population, events that may have occurred outside of the DFWHC area, and out-of-hospital death. Therefore, subsequent validation of these findings is needed in other cohorts.

## Conclusions

When compared with the preimplementation period, use of the protocol was associated with a shorter length of stay in the ED and a higher proportion of patients discharged; there was no difference in the rate of 30-day hospitalization for myocardial infarction or death. This or similar protocols to rapidly rule out MI have the potential to reduce ED overcrowding and improve health care quality while maintaining safety.

## Notes

Supplement. **eFigure 1.** Resource Utilization and Safety Cohorts **eFigure 2.** Parkland Hospital/UT Southwestern protocol for Rapid Myocardial Infarction Rule-out Using the hs-cTnT Assay **eFigure 3.** Median Monthly Dwell Times and Troponin to Disposition Times Pre- and Post-Intervention **eTable.** Odds of Admission vs Discharge and Observation vs Discharge
