# Supplementary material for: Association of a Novel Protocol for Rapid Exclusion of Myocardial Infarction With Resource Use in a US Safety Net Hospital
Source: JAMA Netw Open. 2020 Apr 22;3(4):e203359. doi: 10.1001/jamanetworkopen.2020.3359 (PMC7177202; doi:10.1001/jamanetworkopen.2020.3359)
Supplement: Supplement. — eFigure 1. Resource Utilization and Safety Cohorts eFigure 2. Parkland Hospital/UT Southwestern protocol for Rapid Myocardial Infarction Rule-out Using the hs-cTnT Assay eFigure 3. Median Monthly Dwell Times and Troponin to Disposition Times Pre- and Post-Intervention eTable. Odds of Admission vs Discharge and Observation vs Discharge [file jamanetwopen-3-e203359-s001.pdf]

Supplementary Online Content

Vigen R, Diercks DB, Hashim IA, Pandey A, Zhong L, et al. Association of a novel protocol for rapid exclusion of Myocardial Infarction with resource use in a US Safety Net Hospital. *JAMA Netw Open*. 2020;3(4):e203359. doi:10.1001/jamanetworkopen.2020.3359

eFigure 1. Resource Utilization and Safety Cohorts

eFigure 2. Parkland Hospital/UT Southwestern Protocol for Rapid Myocardial Infarction Rule-out Using the hs-cTnT Assay

eFigure 3. Median Monthly Dwell Times and Toponin to Disposition Times Pre and Post-Intervention

eTable 1. Odds of Admission vs Discharge and Observation vs Discharge

This supplementary material has been provided by the authors to give readers additional information about their work.

© Vigen R et al. *JAMA Netw Open*.**Open Access:** This is an open access article distributed under the terms of the CC-BY License.

eFigure 1: Resource Utilization and Safety Cohorts

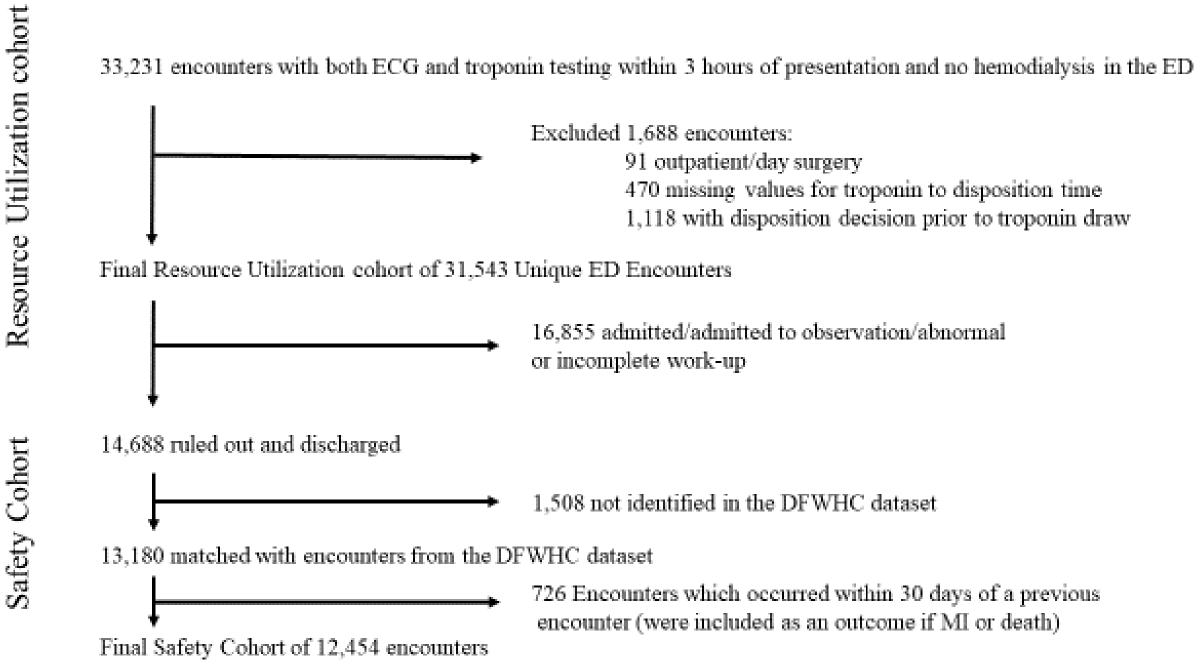

© Vigen R et al. *JAMA Netw Open*.**Open Access:** This is an open access article distributed under the terms of the CC-BY License.

eFigure 2: Parkland Hospital/UT Southwestern protocol for rapid myocardial infarction rule-out using the hs-cTnT assay. CTA = Coronary CT Angiography

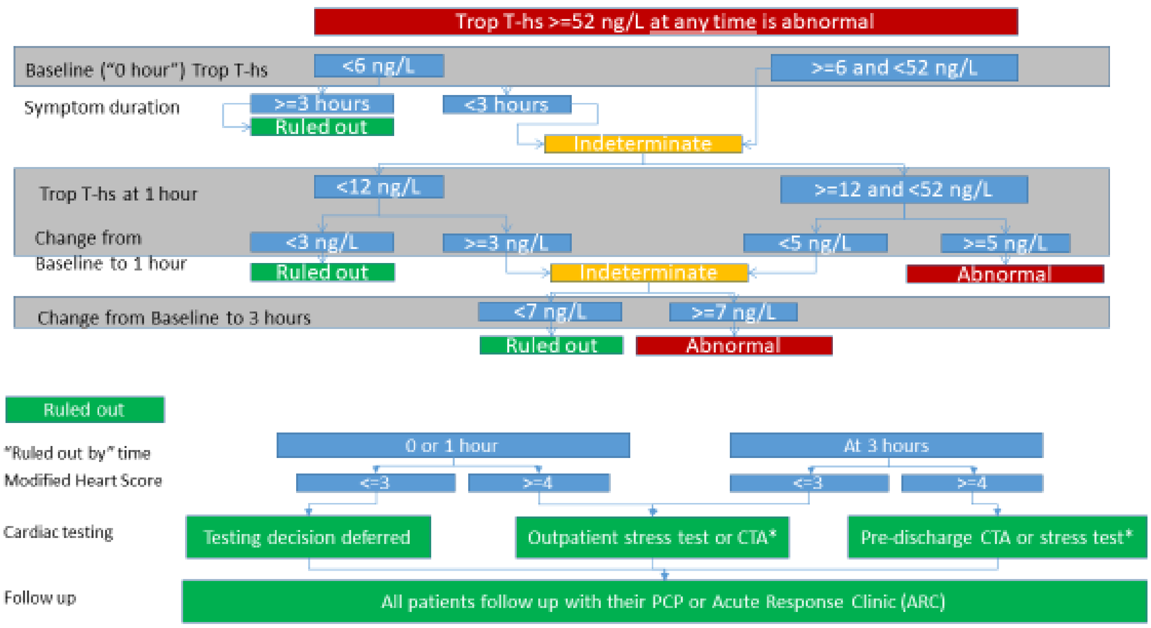

© Vigen R et al. *JAMA Netw Open*.**Open Access:** This is an open access article distributed under the terms of the CC-BY License.

eFigure 3: Median Monthly Dwell Times and Toponin to disposition times pre and post-intervention

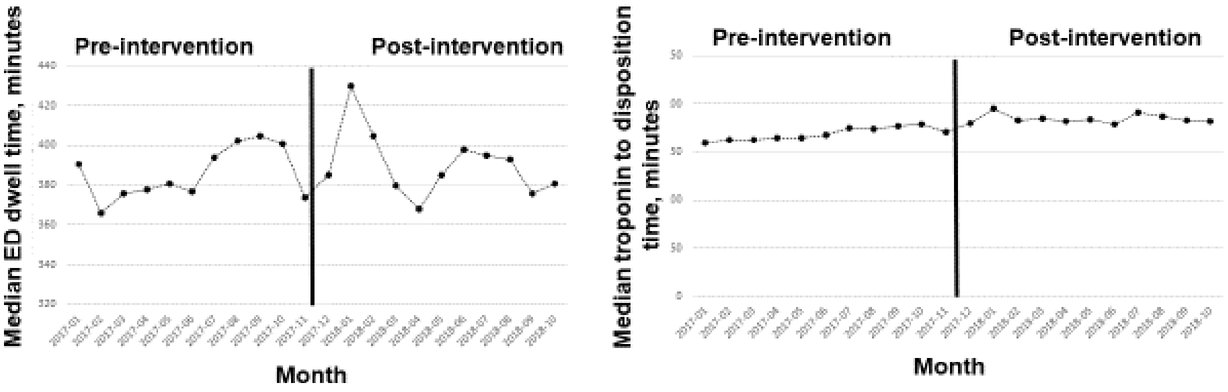

© Vigen R et al. *JAMA Netw Open*.**Open Access:** This is an open access article distributed under the terms of the CC-BY License.

Table 3. Odds of Admission vs Discharge and Observation vs Discharge

	Preintervention		Postintervention		*P* values (interaction term) for OR admit vs discharge	*P* values (interaction term) for OR observe vs discharge
	**OR admit vs discharge**	**OR observe vs discharge**	**OR admit vs discharge**	**OR observe vs discharge**		
**Odds of admission vs discharge and observation vs discharged pre- and postintervention, unadjusted analysis**						
Total cohort (n = 31 543)	0.98 (0.97-1)	0.99 (0.98-1)	0.97 (0.95-1)	1 (0.97-1.03)	.27	.08
Chest pain subcohort (n = 12 845)	0.98 (0.96-1)	0.98 (0.97-1)	0.98 (0.93-1.03)	1.01 (0.97-1.06)	.89	.06
Subcohort with MI excluded (n = 25 611)	0.98 (0.97-0.99)	0.99 (0.98-1)	0.98 (0.94-1.01)	1.01 (0.97-1.04)	.63	.09
Chest pain and MI excluded subcohort (n = 10 917)	0.98 (0.96-1.01)	0.98 (0.97-1)	0.99 (0.93-1.06)	1.01 (0.97-1.06)	.58	.045
**Odds of admission vs discharge and observation vs discharged pre- and post-intervention, adjusted analysis**						
Total cohort (n = 25 082)	0.98 (0.97-0.99)	0.99 (0.97-1)	0.98 (0.95-1.01)	1.01 (0.98-1.04)	.91	.03
Chest pain subcohort (n = 10 420)	0.98 (0.96-1)	0.98 (0.96-1)	0.99 (0.94-1.04)	1.01 (0.97-1.06)	.54	.02
Subcohort with MI excluded (n = 20 505)	0.98 (0.96-0.99)	0.99 (0.98-1)	0.98 (0.95-1.02)	1.01 (0.98-1.05)	.78	.03
Chest pain and MI excluded subcohort (n = 8930)	0.98 (0.96-1)	0.98 (0.96-1)	1 (0.94-1.06)	1.02 (0.97-1.07)	.35	.01

Abbreviation: IQR, interquartile range.

© Vigen R et al. *JAMA Netw Open*.**Open Access:** This is an open access article distributed under the terms of the CC-BY License.
